# Correlational study between SARS-CoV-2 infection and incidence of situs inversus in China

**DOI:** 10.1186/s13023-025-03944-5

**Published:** 2025-08-03

**Authors:** Zhenglong Guo, Ruili Wang, Wenke Yang, Xin Chen, Bingtao Hao, Shixiu Liao

**Affiliations:** 1 Henan Provincial Key Laboratory of Genetic Diseases and Functional Genomics, Medical Genetics Institute of Henan Province, Henan Provincial People’s Hospital, People’s Hospital of Zhengzhou University, No. 7 Weiwu Road, Zhengzhou City, Henan Province China; 2 NHC Key Laboratory of Birth Defects Prevention, Henan Key Laboratory of Population Defects Prevention, Zhengzhou, China

**Keywords:** SARS-CoV2, Pregnancy outcomes, Situs inversus

## Abstract

Wang and colleagues linked SARS-CoV-2 infection during early pregnancy to situs inversus, a rare developmental anomaly of organ placement. However, the absence of genetic testing data raises concerns regarding the reliability of their conclusions, particularly since a similar association was not observed in Scandinavian countries. In this study, we summarize the cases of situs inversus diagnosed during the COVID-19 pandemic in Henan, China, which indicates a significant increase in the incidence of this condition. Among the 34 cases examined, 24 did not reveal any potential pathogenic variants through genetic testing. Our findings suggest a correlation between SARS-CoV-2 infection and the development of situs inversus.

## Objective

Recent studies have established a connection between SARS-CoV-2 infection and adverse pregnancy outcomes, such as miscarriage and preterm birth [1, 2]. A notable publication in the New England Journal of Medicine has highlighted an association between SARS-CoV-2 infection and situs inversus [3], a rare congenital abnormality. This condition includes situs inversus totalis, characterized by a complete mirrored arrangement of all visceral organs, and partial situs inversus, which involves the displacement of specific organs, such as levocardia [3] and heterotaxy syndrome [4]. The pandemic period from December 2022 to February 2023 impacted approximately 82% of the population in China [5]. While these findings provide valuable insights into the potential role of environmental factors in the pathogenesis of situs inversus, the absence of genetic testing data raises concerns about the reliability of the study. Furthermore, it is important to note that a similar increase in situs inversus has not been observed in three Scandinavian countries [6], which may be attributed to the differences in the strains of the coronavirus in the populations of the two regions. In light of these discrepancies, the present study seeks to reevaluate the correlation between SARS-CoV-2 infection and the occurrence of situs inversus in Henan, the third most populous province in China. During the epidemic in question, an estimated 89% of individuals in Henan were infected, with the Omicron BA.5.2 variant being the dominant strain, accounting for approximately 95% of cases.

## Study design

As a leading Prenatal Diagnosis Center responsible for 76 institutions across six southeast regions of Henan, China, we undertook a comprehensive review, aggregating cases to exclude congenital malformations. Our analysis focused on summarizing incidence of fetal situs inversus diagnosed through ultrasonography spanning from 2018 to 2023.

## Results

**Fig. 1:**
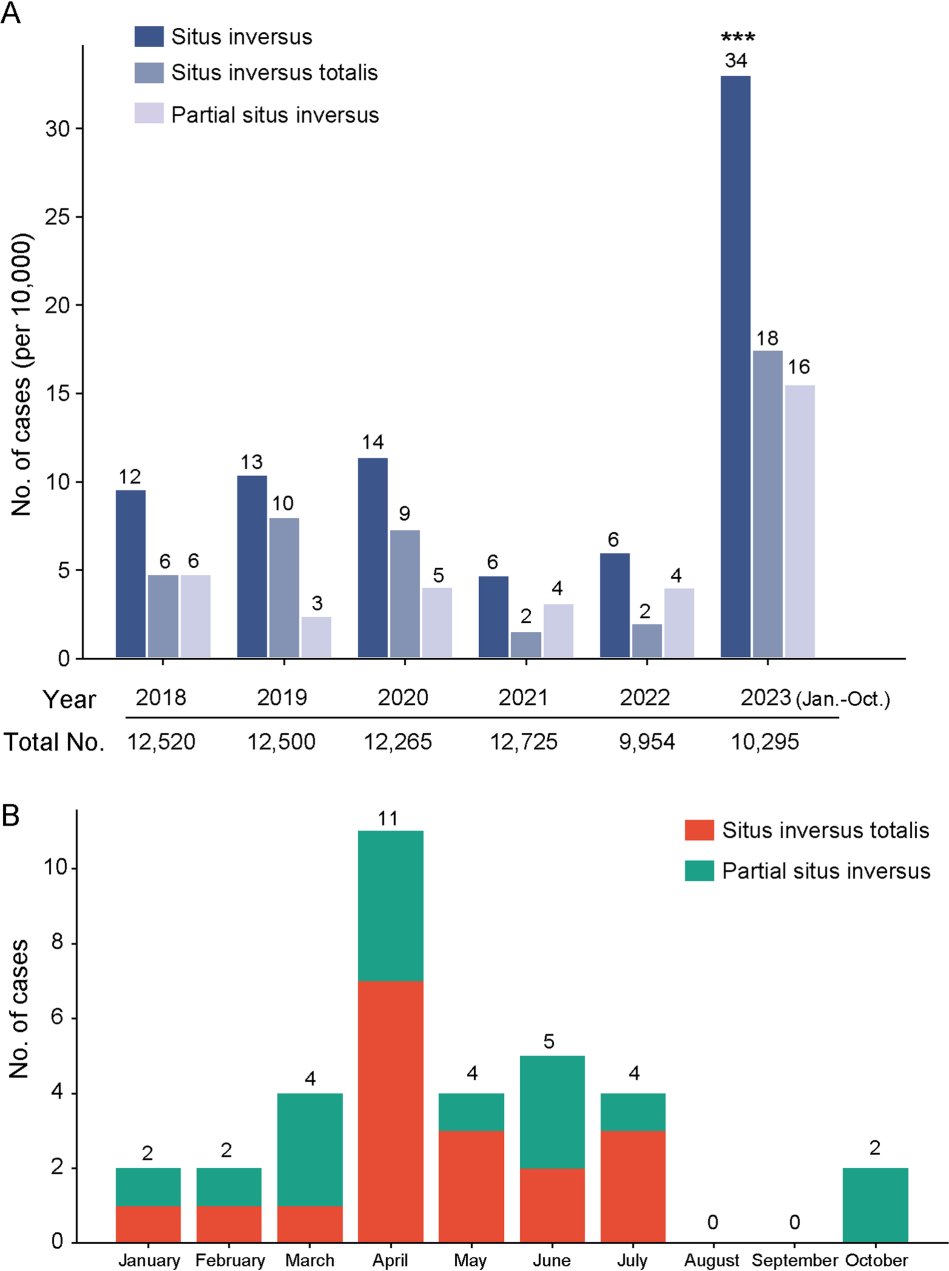
Incidence of Situs Inversus in Henan, China. (**A**) Incidence of fetal situs inversus diagnosed through ultrasonography from January 2018 to October 2023 in Henan Provincial People’s Hospital, China. A total of 34 cases of situs inversus (SI) was identified including 18 cases of situs inversus totalis and 16 cases of partial situs inversus. The Y-axis represents the number of SI cases per 10,000 screened pregnancies. A Chi-square test for difference analysis revealed a significant difference, χ^2^(1) = 43.71, *P* < 0.001. (**B**) Distribution of case numbers of situs inversus in 2023, with the majority occurring from March to July

Consistent with the findings of the previous study [1], we observed that the incidence of fetal situs inversus during the pandemic period (33.0 cases per 10,000 screened pregnancies), as detected through ultrasonography, exhibited an almost fourfold increase compared to the combined incidence over the past five years (8.5 cases per 10,000 screened pregnancies) (Fig. 1). Notably, our study reveals a more pronounced increase in partial situs inversus (12 cases of isolated levocardia and 4 cases of right atrial isomerism [7], a type of heterotaxy syndrome) than in situs inversus totalis. This finding diverges from the observations reported by Wang et al. The discrepancy may be attributed to variations in regional ultrasound diagnostic criteria, which could potentially affect physicians’ sensitivity in detecting situs inversus anomalies. Among the 34 pregnancies with fetal situs inversus, the average gestational age at which pregnant women are diagnosed via ultrasound is approximately 22 weeks, and 28 were confirmed to have SARS-CoV-2 infection through antigen test strip or nucleic acid quantification. Furthermore, our study outcomes revealed that among the 34 identified cases of situs inversus, no pathogenic or likely pathogenic mutations, including variants in currently reported genes such as *NODAL*, *DNAH5*, and *CFAP53* [8], were identified through simultaneous Comparative Genomic Hybridization Microarray (CMA) or/and Whole Exome Sequencing (WES) analyses in 24 cases. While prenatal diagnosis was not performed on the remaining 10 cases, the rate of negative findings is significantly higher than in previous years. A recent study has suggested that the receptor for SARS-CoV-2, ACE2, is co-expressed with key regulatory factors involved in left-right asymmetry during embryonic development. This finding indicates that SARS-CoV-2 infection may directly disrupt visceral lateralization. Additionally, the maternal inflammatory response induced by SARS-CoV-2 may also adversely affect normal fetal development, potentially leading to birth defects [9]. Consequently, we propose that SARS-CoV-2 infections, rather than the currently reported gene variants, may significantly contribute to the pathogenesis of the observed cases of situs inversus.

## Conclusion

This study posits that the observed escalation in fetal situs inversus incidence is plausibly linked to SARS-CoV-2 infection, thus underscoring the conceivable influence of environmental factors in the pathogenesis of situs inversus.

## Data Availability

The data that supports the findings of this study are available in the figure of this article.

## Abbreviations

SARS-CoV-2: Severe Acute Respiratory Syndrome Coronavirus 2
DNAH5: Dynein Axonemal Heavy Chain 5
CFAP53: Cilia and Flagella Associated Protein 53
ACE2: Angiotensin Converting Enzyme 2
